# *Kingella kingae* Intrauterine Infection: An Unusual Cause of Chorioamnionitis and Miscarriage in a Patient with Undifferentiated Connective Tissue Disease

**DOI:** 10.3390/diagnostics11020243

**Published:** 2021-02-04

**Authors:** Maria Paola Bonasoni, Andrea Palicelli, Giulia Dalla Dea, Giuseppina Comitini, Giulia Pazzola, Giuseppe Russello, Graziella Bertoldi, Marcellino Bardaro, Claudia Zuelli, Edoardo Carretto

**Affiliations:** 1 Pathology Unit, Azienda Unità Sanitaria Locale—IRCCS di Reggio Emilia, 42122 Reggio Emilia, Italy; Andrea.Palicelli@ausl.re.it; 2 Pathology Unit, “Maggiore della Carità” Hospital, 28100 Novara, Italy; gdalladea12@gmail.com; 3 Department of Obstetrics & Gynaecology, Azienda Unità Sanitaria Locale—IRCCS di Reggio Emilia, 42122 Reggio Emilia, Italy; Giuseppina.Comitini@ausl.re.it; 4 Rheumatology Unit, Department of Internal Medicine, Azienda USL-IRCCS di Reggio Emilia e Università di Modena e Reggio Emilia, 42122 Reggio Emilia, Italy; Giulia.Pazzola@ausl.re.it; 5 Clinical Microbiology Laboratory, Azienda Unità Sanitaria Locale—IRCCS di Reggio Emilia, 42122 Reggio Emilia, Italy; Giuseppe.Russello@ausl.re.it (G.R.); Graziella.Bertoldi@ausl.re.it (G.B.); Marcellino.Bardaro@ausl.re.it (M.B.); Claudia.Zuelli@ausl.re.it (C.Z.); Edoardo.Carretto@ausl.re.it (E.C.)

**Keywords:** *Kingella kingae*, preterm premature rupture of membrane, Chorioamnionitis undifferentiated connective tissue disease

## Abstract

*Kingella kingae* is a Gram-negative coccobacillus belonging to the *Neisseriaceae* family. In children less than 4 years old, *K. kingae* invasive infection can induce septic arthritis and osteomyelitis, and more rarely endocarditis, meningitis, ocular infections, and pneumonia. In adults, it may be a cause of endocarditis. To date, *K. kingae* acute chorioamnionitis (AC) leading to preterm rupture of membranes (PPROM) and miscarriage has never been reported. Herein, we describe a case of intrauterine fetal death (IUFD) at 22 weeks’ gestation due to *K. kingae* infection occurred in a patient affected by undifferentiated connective tissue disease (UCTD) in lupus erythematosus systemic (LES) evolution with severe neutropenia. *K. kingae* was isolated in placental subamnionic swab and tissue cultures as well as fetal ear, nose, and pharyngeal swabs. Placental histological examination showed necrotizing AC and funisitis. In the fetus, neutrophils were observed within the alveoli and in the gastrointestinal lumen. Maternal medical treatment for UCTD was modified according to the *K. kingae* invasive infection. In the event of IUFD due to AC, microbiological cultures on placenta and fetal tissues should always be carried out in order to isolate the etiologic agent and target the correct medical treatment.

## 1. Introduction

*Kingella kingae* is a Gram-negative, facultatively anaerobic coccobacillus of the Neisseriaceae family. It is a slowly growing bacterium also a member of the HACEK group (*Haemophilus* spp., *Aggregatibacter actinomycetemcomitans*, *Cardiobacterium hominis*, *Eikenella corrodens*, and *Kingella kingae*) [1,2]. *K. kingae* is currently recognized as the most common etiology of septic arthritis and osteomyelitis in children between the ages of 6 and 48 months. More rarely, it can cause complicated endocarditis, meningitis, ocular infections, pericarditis, peritonitis, and pneumonia [3,4].

To the best of our knowledge, *K. kingae* infection has never been reported in pregnancy. Only a case of early onset sepsis (EOS) in a premature infant has been described [5]. Herein, we present an unusual case of preterm premature rupture of membranes (PPROM) at 22 weeks’ gestation with subsequent intrauterine fetal death (IUFD) due to severe acute chorioamnionitis (AC). Placental subamnionic swab and tissue microbiological cultures isolated *K. kingae*, including fetal ear, nose, and pharyngeal swabs. Moreover, the 31-year-old mother was affected by undifferentiated connective tissue disease (UCTD) in evolution toward lupus erythematosus systemic (LES); severe neutropenia, antinuclear antibodies (ANA), and low titer of anti-neutrophil cytoplasmic antibodies (ANCA) were also present.

## 2. Case Description

### 2.1. Mother Clinical Presentation and Treatment

A 31-year-old mother, gravida 2 para 1, presented at 22 weeks’ gestation to the Emergency Department of our Institution for miscarriage. The patient had been suffering from shaking chills, fever (38 °C), stomatitis, sore throat, abdominal pain, and minimal vaginal bleeding for the previous three days. Gynaecological examination revealed on speculum mild fresh blood in vagina and abdominal ultrasound (US) observed IUFD. She was admitted to the hospital, labour was induced and a stillborn female fetus was delivered. Placental microbiological samples and fetal swabs were sent to the Clinical Microbiology laboratory where cultures on different media were immediately performed.

Overall, the patient had a complex clinical history. She had suffered from monoarticular juvenile idiopathic arthritis, completely resolved at 15 years of age.

Six years before the current miscarriage, she had complained of bilateral gonalgia. Laboratory findings had shown severe neutropenia (250 neutrophils) and low titer of ANCA. Antinuclear antibodies (ANA) titer had been 1:160. Anti extractable nuclear antigens (ENA), anti-DNA antibodies, and antiphospholipid antibodies (lupus anticoagulant—LAC; anticardiolipin antibodies—aCL; anti-β2-glycoprotein-1—anti-β2GP-1) had been negative. Bone marrow biopsy had been reported within normal limits.

In the previous six years, although the patient had always been presenting with low neutrophils, she had never contracted opportunistic infections.

One year before the miscarriage, she had had her first pregnancy with a vaginally delivered male infant of 3200 g at 40 weeks + 6 days of gestational age. One month after delivery, she had severe mastitis, which required hospital admission and surgical treatment. After that, she presented with two episodes of bilateral knee arthritis, and then she was put on hydroxychloroquine 200 mg twice a day (tablets).

Regarding the current miscarriage, at hospital admission, maternal laboratory findings were as follows: WBC 2.6 x 1.000/μL and C-reactive protein (CRP) 13.45 mg/dL. During the first day after IUFD, CRP worsened to 18.02 mg/dL, then progressively decreased from 16.46 (2 days), 2.7 mg/dL (3 days), 1.43 mg/dL (5 days) to 0.11 mg/dL (10 days). Blood cultures performed at admission resulted negative.

Maternal medical treatment, started soon after IUFD, consisted of 2 days of meropenem, subsequently switched to piperacillin/tazobactam for 6 days. She fully recovered and was discharged after 10 days.

After hematologic and rheumatologic consult, due to low neutrophil count and recent intrauterine infection, Granulocyte-Colony Stimulating Factor (G-CSF) and cyclosporine (200 mg a day) were added to hydroxychloroquine.

### 2.2. Fetal Autopsy and Microbiological Results

**Figure 1:**
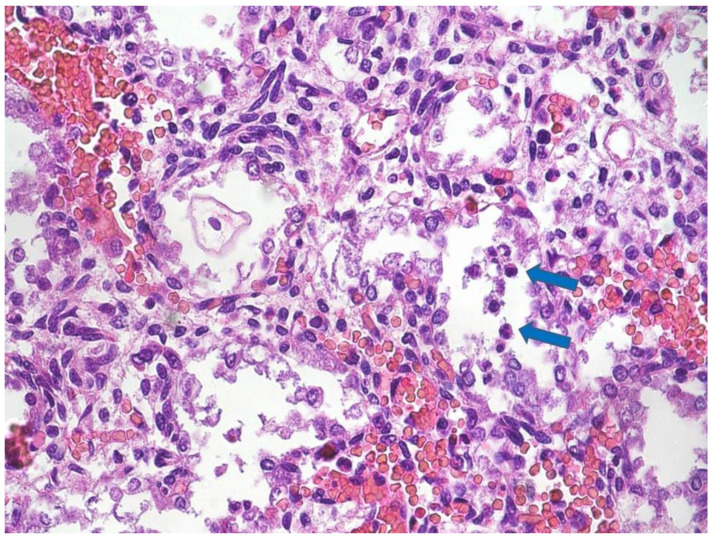
Fetal lung: A few neutrophils within the alveoli (blue arrows; Hematoxylin Eosin (HE) staining 20×).

Postmortem examination revealed a nonmacerated female fetus weighing 420 g and measuring 29 cm in crown-heel length. The other measurements were as follows: Crown-rump length, 19.5 cm; foot length, 3.7 cm; head, chest and abdominal circumference, 18, 16, and 15 cm, respectively. Overall, anthropometric measurements were consistent with 22 weeks’ gestation [6]. External examination showed a normal fetus with mild eyelid and nuchal oedema. On internal examination, intestinal rotation was incomplete (malrotation) with short midgut mesenteric attachment and mobile intestine. No volvulus was observed. Microscopic analysis revealed the presence of intra-alveolar, gastric and intestinal neutrophils (Figure 1); oedema, microhemorrhages and acute tubular necrosis (NTA) of the renal parenchyma were also present.

**Figure 2:**
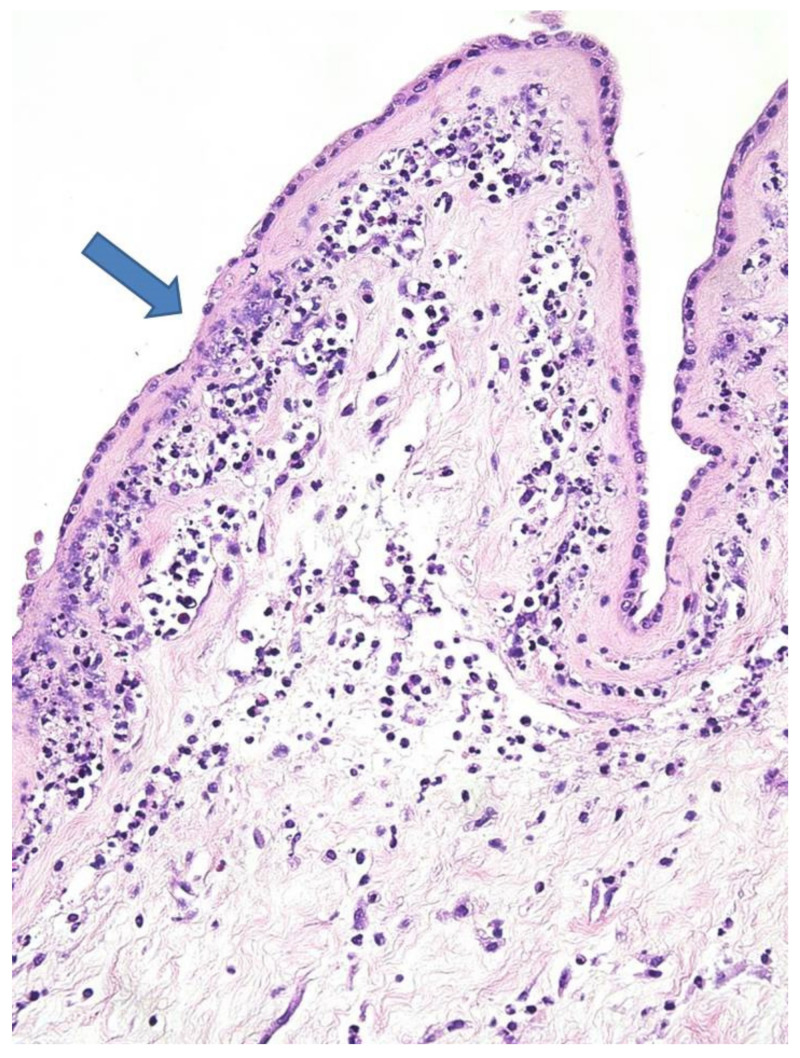
Amniochorial membranes: Severe chorioamnionitis with amnion necrosis (blue arrow; HE staining 10×).

**Figure 3:**
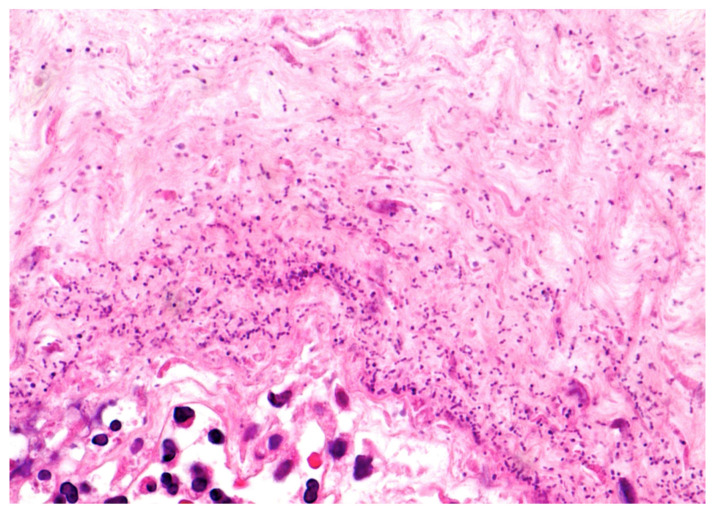
Chorion: Abundant coccoid bacteria within the chorionic stroma (HE staining 40×).

**Figure 4:**
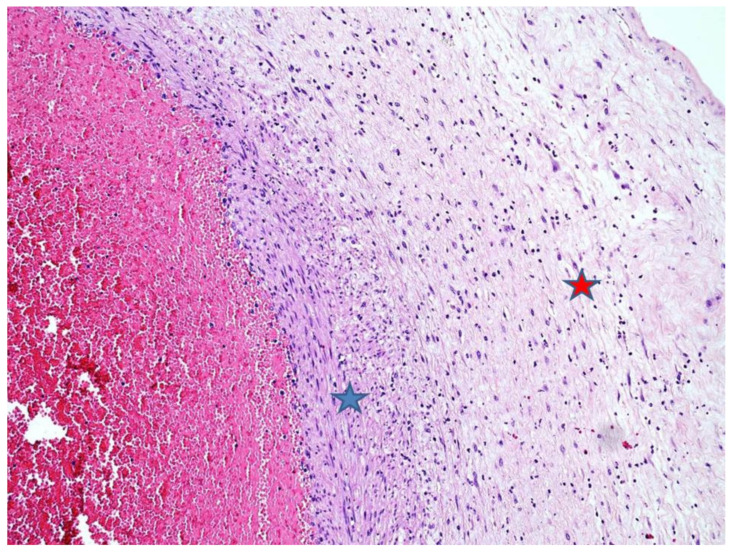
Umbilical artery acute vasculitis: Neutrophils within the arterial wall (blue star) with extension to Warthon’s jelly (red star) (HE staining, 4×).

The placenta was received complete, weighed 137 g, and measured 10 × 10 × 2 cm. The membranes were yellowish and opaque. Microscopically, there was necrotizing AC (Figure 2) corresponding to a maternal inflammatory response stage 3/3 and grade 2/2 [7]. Coccoid bacteria were noted at high magnification within the chorion (Figure 3). Funisitis was also observed with neutrophilic infiltrate of the umbilical vein, and the two arteries with extension to Wharton’s jelly (Figure 4). These findings were consistent with fetal inflammatory response stage 2/3 and grade 2/2 [7]. In addition, in the placental parenchyma some recent infarcts were detected, and in the decidua few spiral arteries presented initial thrombosis.

Autoptical samples were cultured on Columbia blood agar (CBA), McConkey agar (MCA), mannitol salt agar (MSA) and Sabouraud agar (SAB; all these media were incubated at 36 °C, ambient air), chocolate agar (CA, incubated at 36 °C, 5% CO_2_), and Schaedler agar (SA, incubated at 36 °C, anaerobic atmosphere). After 48 hours from the fetus’s pharyngeal, nose, and ear swabs, small colonies grew on CBA and CA (better on the latter). No growth was observed on MCA. These smooth colonies were catalase negative and oxidase positive. They were identified as *K. kingae* using the MALDI-ToF technology (Bruker Daltonics, Germany), Biotyper OC software version 3.1. Microbiological cultures on fetal blood and tissues (lung and liver) showed no growth after 72 hours and were discarded as negative. Instead, *K. kingae* also grew on subamniotic swab and placental tissue cultures. If present, the microorganism grew as a pure culture in all the samples. Antimicrobial susceptibility testing was not performed.

## 3. Materials and Methods

We searched for (*Kingella kingae* AND (chorioamnionitis OR genital OR amniotic OR amnios OR placenta OR placental OR funisitis OR fetus OR fetal OR pregnancy OR pregnant OR (membrane AND rupture) OR uterus OR uterine OR intrauterine)) in Pubmed (all fields, 2 results), Scopus (Title/Abstract/Keyword, 9 results) and Web of Science (Topic/Title, 2 results). No limitations were set. The bibliographic research ended on 25 December 2020. Globally, a total of 10 articles resulted from our search. Titles and Abstract of all the articles were screened. All articles were excluded as non-relevant.

## 4. Discussion

*K. kingae* is a Gram-negative coccobacillus belonging to the Neisseriaceae family. It is a normal component of the oropharyngeal flora of children less than 4 years old. However, the bacterium, after having colonized and breached the epithelial surface, may disseminate in the bloodstream causing, especially in children, arthritis, osteomyelitis, and endocarditis. This latter complication may also occur in adults [1,8].

Mechanisms of virulence include biofilm formation, pili, RTX toxin production, polysaccharide capsule, and secretion of outer membrane vesicles (OMV).

*K. kingae* is able to form biofilms, which consist of large quantities of bacteria clustered together in a polysaccharide “slime”, that tightly attaches to the mucosal surface. This kind of colonization allows bacteria to survive in a protected environment preserving them from dehydratation, immune response, and antibiotics [9,10].

*K. kingae* anchors to the epithelia thanks to type 4 pili, which are proteinaceous fimbriae necessary for efficient adherence to respiratory mucosa and synovia [11].

Moreover, RTX toxin expression exerts a wide-spectrum cytotoxic effect on macrophages, leukocytes, synoviocytes, and respiratory epithelial cells. This toxin plays a key role in infection spreading favouring epithelial disruption and bloodstream dissemination [12,13]. Of note, type a and b polysaccharide capsule predominate in invasive isolates [14]. A further virulence factor possessed by *K. kingae* is the secretion of OMV, which are small parts of the outer membrane encasing periplasm proteins that bulge away from the bacterium and then are released in the extracellular space. These OMV’s are hemolytic and leukotoxic in an in vitro model and internalized by synoviocytes inducing the synthesis of granulocyte-macrophage colony stimulating factor (GM-CSF) and interleukin 6 (IL-6), likely representing the in vivo immunitary response in joint infections [15].

*K. kingae* has been recognized as a leading cause of septic arthritis and osteomyelitis in children less than 4 years old, usually with no underlying medical conditions [3,4]. Other rare primary manifestations in pediatric population are: Pneumonia, endocarditis, soft tissue infection, endophtalmitis, orbital cellulitis, and meningitis [1,2,16,17,18,19,20].

Being part of the HACEK group (*Haemophilus* spp, *Aggregatibacter actinomycetemcomitans*, *Cardiobacterium hominis*, *Eikenella corrodens*, *Kingella kingae*), *K. kingae* can cause endocarditis, affecting more frequently children and young adults [21].

Adult patients usually have predisposing conditions in order to develop invasive *K. kingae* disease, such as malignancies, liver cirrhosis, diabetes, sickle cell anemia, and renal transplant [1,22,23,24,25].

However, to the best of our knowledge, *K. kingae* infection has never been reported in pregnancy and besides as a cause of PPROM and miscarriage. Moreover, the patient was affected by UCTD in evolution toward LES with severe neutropenia, ANA, and ANCA.

The 31-year-old mother presented to the hospital after having suffered shaking chills, fever (38 °C), stomatitis, sore throat, abdominal pain, and minimal vaginal bleeding for the previous three days. US revealed IUFD. Microbiological cultures on placental subamnionic swab and parenchymal tissue isolated *K. kingae*. The same agent grew on fetal swabs (ear, nose, and pharyngeal). Fetal tissue cultures (blood, lung and liver) resulted negative. Placental histological examination confirmed severe necrotizing chorioamnionitis and funisitis. Within the chorion, coccoid bacteria were also observed. Fetal histology showed few neutrophils within the alveoli, and gastro-intestinal lumen, respectively.

*K. kingae* was then considered the etiologic agent responsible for the miscarriage. While other members of the genus *Kingella* such as *K. negevensis* and *K. denitrificans* are known to cause bacterial vaginosis, chorioamnionitis, and pediatric vaginitis [26,27,28]; *K. kingae* infections of the urogenital tract are extremely rare [29].

However, AC due to *K. kingae* has never been described. The main microorganisms responsible for AC usually are group B *Streptococcus*, *Fusobacterium nucleatum*, *Peptostreptococcus*, *Escherichia coli*, *Bacteroides* species, *Ureaplasma urealyticum*, and *Listeria monocytogenes* [30,31]. As *K. kingae* is not part of the lower genital tract flora, in the case described, an ascending infection was unlikely. The bacterium probably reached the amniotic cavity through the hematogenous pathway from the oropharynx. In fact, the mother presented with sore throat and stomatitis. Although reported in children, *K. kingae* invasive disease has been associated with recent or concomitant virus infections like coxsackievirus, herpes simplex, varicella zoster, and rhinovirus in the oropharynx and upper respiratory tract [32,33,34,35]. Therefore, a concomitant viral infection in those locations may represent a cofactor in the pathophysiology of invasive *K. kingae*, probably favoring epithelial breaching and altering the local immune response [36].

In the case we described, it is impossible to differentiate a condition of maternal oropharyngeal carrier exacerbated by a concomitant viral infection or a *K. kingae* primary oropharyngeal infection turned out to be invasive. Notwithstanding, the mother was affected by UCTD in evolution toward LES with severe neutropenia. To date, *K. kingae* infection has never been described in this kind of peculiar setting. Only few cases have been observed in a granulocytopenic host [22], LES [37,38,39,40], rheumatoid arthritis [41], and acquired immunodeficiency syndrome (AIDS) [42,43,44,45,46].

Besides, recognition of *K. kingae* intrauterine infection allowed modifying maternal therapeutic plan, as G-CSF and cyclosporine were added to hydroxychloroquine.

In our specific case, placental cultures and fetal swabs revealed *K. kingae* infection, but the microorganism was not retrieved from fetal blood and tissues. This may be explained as a low bacterial load or a possible specimen type inhibition. In fact, it is known that recovery of *K. kingae* from bacteriological solid media may be difficult and nucleic acid amplification is advisable [1]. In our case, polymerase chain reaction (PCR) on the fetal tissues was not performed as infection was clearly demonstrated by neutrophils within the alveoli and in the lumen of gastrointestinal tract.

Usually, in the event of miscarriage due to chorioamnionitis, identification of the correct etiologic agent is of paramount importance as maternal medical treatment can be adjusted accordingly. Placental subamniotic swab and parenchymal cultures as well as fetal blood and tissues microbiological studies should always be carried out as recommended by perinatal autopsy protocols [47,48].

## Data Availability

The data presented in this study are available on request from the corresponding author.
